# Krüppel-Like Factor 6 Is Required for Oxidative and Oncogene-Induced Cellular Senescence

**DOI:** 10.3389/fcell.2019.00297

**Published:** 2019-11-22

**Authors:** Maria Eugenia Sabatino, Andrés Castellaro, Ana C. Racca, Sofía Carbajosa González, Maria Florencia Pansa, Gastón Soria, Jose Luis Bocco

**Affiliations:** ^1^Departamento de Bioquímica Clínica, Facultad de Ciencias Químicas, Universidad Nacional de Córdoba, Córdoba, Argentina; ^2^Centro de Investigaciones en Bioquímica Clínica e Inmunología (CIBICI), CONICET, Universidad Nacional de Córdoba, Córdoba, Argentina

**Keywords:** KLF6, cellular senescence, DNA damage, cell proliferation, ras oncogene

## Abstract

Krüppel-like factor 6 (KLF6) is a transcription factor involved in the regulation of several cellular processes. Regarding its role in tumorigenesis, KLF6 is considered a tumor suppressor. Numerous reports demonstrate its frequent genomic loss or down-regulation, implying a functional inactivation in a broad range of human cancers. Previous work from our laboratory showed that the down-regulation of KLF6 expression in normal fibroblasts leads to cellular transformation, while its ectopic expression interferes with the oncogenic transformation triggered by activated Ras through a cell cycle arrest. We hypothesize that the growth suppressor activity of KLF6 may involve the induction of cellular senescence thereby helping to prevent the proliferation of cells at risk of neoplastic transformation. Here, we explored the association of KLF6 up-regulation in two different cellular senescence scenarios. We found that KLF6 silencing bypasses both oxidative and oncogene-induced senescence. In this context, KLF6 expression *per se* was capable to trigger cellular senescence in both normal and tumoral contexts. As such, the findings presented in this report provide insights into a potential mechanism by which KLF6 may play a suppressing role of uncontrolled or damaged cell proliferation.

## Introduction

Krüppel-like factor 6 (KLF6) belongs to a zinc-finger-containing transcription factors family and has been implicated in the regulation of several cellular processes including development, proliferation, inflammation, apoptosis, and differentiation (Bieker, 2001; Ito et al., 2004; Matsumoto et al., 2006; DiFeo et al., 2009; McConnell and Yang, 2010; Racca et al., 2011; Racca et al., 2015). KLF6 transcription factor has a growing list of target genes, many of which are involved in cell cycle regulation (Tetreault et al., 2013). For example, KLF6 up-regulates cyclin-dependent kinase (CDK) inhibitors such as p21^WAF1/Cip1^ (Narla et al., 2001; Narla et al., 2007) and interacts with other transcription factors as Sp1, KLF4, and p53 (Rubinstein et al., 2004; DiFeo et al., 2009). KLF6 is reputed as tumor suppressor since numerous reports demonstrate its frequent genomic loss or down-regulation, implying a functional inactivation, in a broad range of human cancers such as glioblastoma, hepatocellular carcinoma, gastric carcinoma, colorectal, prostate, ovarian, breast, and non-small-cell lung cancers (Narla et al., 2001; Kimmelman et al., 2004; Kremer-Tal et al., 2004; Narla et al., 2007; Bureau et al., 2009; DiFeo et al., 2009; Tetreault et al., 2013). In addition, previous reports from our laboratory showed that KLF6 down-regulation leads to spontaneous transformation of NIH3T3 fibroblasts (Trucco et al., 2014). Also, KLF6 ectopic expression impaired H-Ras^G12V^-mediated cell growth and transformed phenotype (Trucco et al., 2014).

Within the strategies to limit cell growth in cells with uncontrolled proliferation, cellular senescence is now believed to play a critical role in the suppression of tumorgenesis in various organs (Campisi, 2001; Braig et al., 2005; Bartkova et al., 2006; Prieur and Peeper, 2008; Vizioli et al., 2011; Sabatino et al., 2015). Cellular senescence involves cell cycle arrest, resulting in a stable loss of proliferative capacity. This can be elicited through various genotoxic stimuli, including telomere dysfunction, oncogenes activation, DNA damage and oxidative stress (Serrano et al., 1997; Lee et al., 2006; Campisi and d’Adda di Fagagna, 2007; Mallette et al., 2007). This cellular program is achieved primarily by the activation of senescence-associated genes such as p53 and pRb (Di Leonardo et al., 1994; Campisi, 2001), which are frequently mutated in a large number of cancers (Burkhart and Sage, 2008; Levine and Oren, 2009). The presumed role of KLF6 as tumor suppressor comes from its ability to reduce cell proliferation through different cellular mechanisms as the regulation of cell cycle components, oncogene signaling and apoptosis (Ito et al., 2004; Kimmelman et al., 2004; Narla et al., 2007). However, it is currently unknown whether KLF6 participates in the induction and/or maintenance of cellular senescence. Given KLF6 ubiquitous expression and its capability to suppress cell growth, we hypothesized that KLF6 may be involved in the induction of cellular senescence mechanism, preventing the proliferation of cells at risk of neoplastic transformation.

Results presented in this report revealed that KLF6 silencing interfered with oxidative and oncogene-induced cellular senescence in NIH3T3 fibroblasts. Additionally, ectopic KLF6 expression was able to induce cellular senescence in normal and tumoral cells. These findings provide evidence for KLF6 involvement in the molecular basis underlying the induction and maintenance of cellular senescence.

## Materials and Methods

### Cell Culture

All cell lines were obtained from the American Type Culture Collection (ATCC, Manassas, VA, United States). NIH3T3 and HeLa cells were cultured in Dulbecco’s modified Eagle medium (DMEM; Gibco, Carlsbad, CA, United States) supplemented with 10% newborn calf serum (Gibco) and penicillin/streptomycin (P3032 and S9137, Sigma-Aldrich, St. Louis, MO, United States). To trigger oxidative-induced cellular senescence, murine NIH3T3 fibroblasts were treated with H_2_O_2_ (150 μM for 2 h in 0.5% newborn calf serum) and processed after 6 days (cultured in 2.5% newborn calf serum) for detection of SA-β-Gal senescence marker or DDR markers (p53, p21, γH2AX, and pATM) or KLF6 expression. Cell lines stably transduced to express a constitutively active form of Ras (H-Ras^G12V^) or KLF6 under the control of a tetracycline-inducible promoter were maintained in DMEM containing 10% Tet system approved fetal bovine serum (#631106, Clontech, Mountain View, CA, United States) and treated with tetracycline (0.1 μg/mL and 1.0 μg/mL, #87128, Sigma-Aldrich) during 6 days and processed as described above for H_2_O_2_ treatment. NIH3T3 and HeLa cells were mycoplasma-free as determined by both, Hoechst staining and PCR.

### Establishment of Stable Cells

Lentiviruses were generated as described in Trucco et al. (2014) by cotransfecting HEK293T cells with pCMV-dR8.2- vpr and pCMV-VSV-G helper plasmids and the appropriate transfer expression vector (pLKO.1-SCR, pLKO.1-shKLF6-A, pLKO.1-shKLF6-B, pLenti3.3/TR, pLenti- CMV/TO/RasV12-Puro, pLenti6.3/TO/RasV12, pLenti6.3/- TO/KLF6). The 21-mer target sequences were scrambled (SCR), 5′-GTTAACTGCGTACCTTGAGTA; shKLF6-A, 5′-ACAGGGAATCTTCTCAACTAT; and shKLF6-B, 5′-GATCC CATTGGTGAAGTCTTA. Viral supernatants were collected 48 h after transfection, supplemented with 6 μg/ml polybrene (sc-134220, Santa Cruz Biotechnology, Santa Cruz, CA, United States), and used for transduction of NIH3T3 cells (multiplicity of infection, 0.5). After 48 h, the cells were resuspended in fresh medium containing puromycin (P-8833, Sigma-Aldrich, St. Louis, MO, United States) or blasticidin (R21001, Thermo Fisher Scientific, Waltham, MA, United States) and selected during 3 weeks. Human cervix cancer HeLa cells stably modify as previously described (D’Astolfo et al., 2008) to express KLF6 under the control of a tetracycline-inducible promoter were treated with 0.1 and 1.0 μg/mL of doxycycline (D9891, Sigma-Aldrich) during 6 days and processed.

### Western Blot Analysis

Western blot analysis was performed as previously described (Racca et al., 2011) and the following antibodies were used: rabbit polyclonal anti-H-Ras (C-20, sc-520, Santa Cruz), mouse monoclonal anti-KLF6 [clone 2c11, previously specified by Gehrau et al. (2011)], rabbit and goat polyclonal anti-p53 (sc6243, Santa Cruz Biotechnology), mouse anti- α -tubulin (T9026, Sigma-Aldrich), mouse anti-β-actin (A5316, Sigma-Aldrich) and donkey-anti-mouse near-Infrared fluorescent secondary antibodies 680CW and 800CW (LI-COR Biosciences, Lincoln, NE, United States). α-tubulin and β-actin were used as loading control. Fluorescence emission was acquired with Odyssey CLx scan (LI-COR, United States). Images are representative of three independent experiments.

### Immunofluorescence Detection

Fluorescence immunodetection was carry on as previously described (Sabatino et al., 2013) and the following antibodies were used: rabbit or goat polyclonal anti-p53 (sc6243, Santa Cruz Biotechnology), mouse monoclonal anti-phospho-Histone γH2AX (Ser139) (#2577, Cell Signaling Technology, Beverly, MA, United States), mouse monoclonal anti-p21^Waf1/Cip1^(2947S, Cell Signaling Technology) and mouse monoclonal anti-ATM (phospho S1981) antibody (ab36810, Abcam, Cambridge, United Kingdom), Alexa Fluor 488 donkey anti-mouse or anti-rabbit (Jackson Immunoresearch, West Grove, PA, United States). Nuclei area is represented by yellow contours extracted from Hoechst fluorescence staining. Micrographs were obtained with a Leica Microscope (Leica DMi8, Leica Microsystems, Wetzlar, Germany) at X630 magnification and are representative of three independent experiments. Fluorescence intensity of each antibody fluorochrome emission was evaluated in five random chosen fields and normalized by total DNA content area.

### SA-β-Gal Detection Assay

To detect senescence associated-β-galactosidase (SA-β-Gal) staining (cytoplasmic blue stain) as previously described (Debacq-Chainiaux et al., 2009) using X-Gal (X-Gal-RO, Sigma-Aldrich). Nuclear fluorescent dye Hoechst was applied to denote cell nuclei (gray stain). Images were captured with Fluoview 1000 (Olympus, Tokyo, Japan) at X400 magnification and are representative of three independent experiments. Image processing was performed with ImageJ software (National Institutes of Health, Bethesda, MD, United States). Cellular senescence index expressed as the percentage of SA-β-Gal positive cells and was obtained counting a minimum of 500 cells on randomly chosen fields from three independent experiments.

### Statistical Analysis

Data are presented as media ± SEM. Unless otherwise reported, one-way ANOVA was used for three independent samples with normal distribution (Wilk–Shapiro test) and homoscedasticity (Levene test). Where data did not meet the assumptions of the test, Generalized Linear Mixed Model (GLMM) test was used (Di Rienzo and Re Casanoves, 2017). Fisher Test was applied to detect significant difference, *p* < 0.05 using InfoStat software (Grupo InfoStat, Facultad de Ciencias Agropecuarias, Universidad Nacional de Córdoba, Córdoba, Argentina).

## Results

### KLF6 Expression Is Induced Upon Oxidative and Oncogene-Induced Cellular Senescence

**FIGURE 1:**
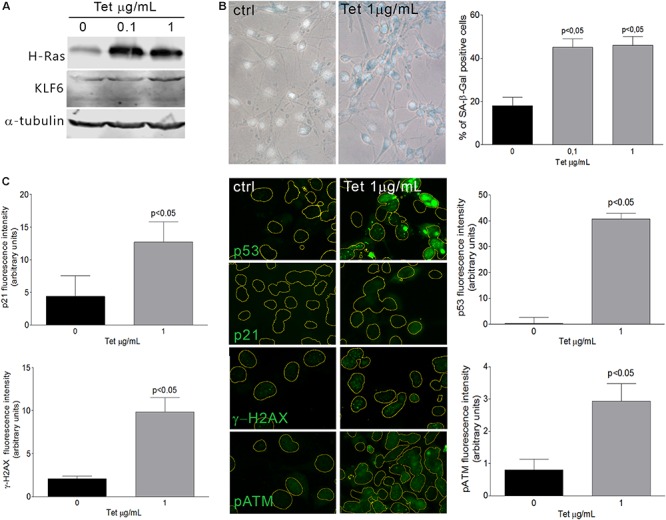
Oncogene-induced senescence in NIH3T3 fibroblasts expressing H-Ras^G12V^. **(A)** Immunoblotting from murine NIH3T3 fibroblasts expressing H-Ras^G12V^ after 3 days of tetracycline treatment (0.1 and 1.0 μg/mL). Anti-α-tubulin was used as loading control. Images are representative of three independent experiments. **(B)** Left: Representative micrograph of murine NIH3T3 fibroblasts stably transduced to express a constitutively active form of Ras (H-Ras^G12V^) under the control of a tetracycline-inducible promoter. Cells were treated with tetracycline (0.1 and 1.0 μg/mL) during 6 days and processed to detect senescence associated-β-galactosidase (SA-β-Gal) staining (cytoplasmic blue stain). Nuclear fluorescent dye Hoechst was applied to denote cell nuclei (gray stain). Images were captured at X400 magnification and are representative of three independent experiments. Right: Cellular senescence index expressed as the percentage of SA-β-Gal positive cells in NIH3T3 fibroblast expressing H-Ras^G12V^. **(C)** Representative micrograph of DNA damage response biomarkers: p53, p21, γ-H2AX, and phospho ATM by fluorescence immunodetection on murine NIH3T3 fibroblasts expressing H-Ras^G12V^ under tetracycline control. Nuclei area is represented by yellow contours extracted from Hoechst fluorescence staining at X630 magnification. Images are representative of three independent experiments. Fluorescence intensity of each antibody fluorochrome emission was evaluated in five random chosen fields and normalized by total DNA content area. Data are presented as media ± SEM. Fisher Test was applied to detect significant difference, *p* < 0.05.

**FIGURE 2:**
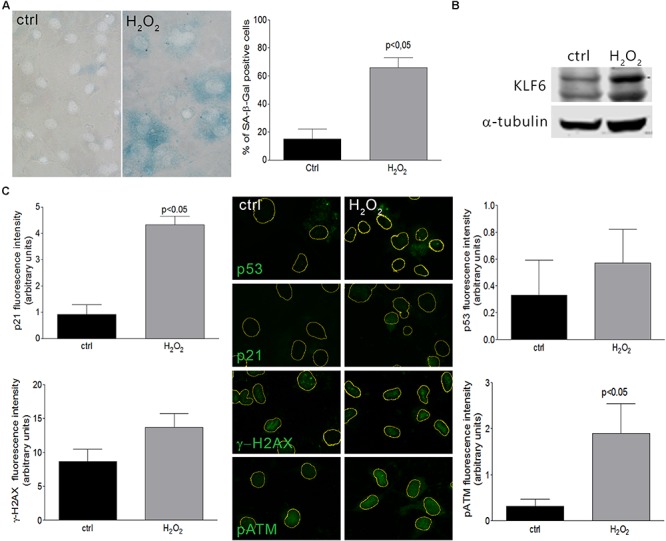
Cellular senescence in NIH3T3 fibroblasts treated with H_2_O_2_. **(A)** Left: Representative micrograph of murine NIH3T3 fibroblasts treated with H_2_O_2_ (150 μM for 2 h) and processed after 6 days to detect SA-β-Gal staining (cytoplasmic blue stain). Nuclear fluorescent dye Hoechst was applied to denote cell nuclei (gray stain). Images were captured at X400 magnification and are representative of three independent experiments. Right: Cellular senescence index expressed as the percentage of SA-β-Gal positive cells. **(B)** KLF6 immunoblotting from murine NIH3T3 fibroblasts after 4 h of H_2_O_2_ treatment (150 μM for 2 h). Anti-α-tubulin was used as loading control. Images are representative of three independent experiments. **(C)** Representative micrograph of DNA damage response biomarkers: p53, p21, γ-H2AX and phospho ATM by fluorescence immunodetection on murine NIH3T3 fibroblasts treated with H_2_O_2_. Images are representative of three independent experiments. Fluorescence intensity of each antibody fluorochrome emission was evaluated in five random chosen fields and normalized by total DNA content area. Data are presented as media ± SEM. Fisher Test was applied to detect significant difference, *p* < 0.05.

Cellular senescence phenomenon is usually detected by the elevation of senescence-associated β-galactosidase (SA–β-Gal) enzyme activity (Dimri et al., 1995; Lee et al., 2006). Additionally, senescent phenotypes usually correlate with the accumulation of DNA damage markers such as γ-H2AX (histone γ-H2AX) and pATM (phosphorylated Ataxia Telangiectasia Mutated) (Di Micco et al., 2006), as well as the activation of p53 or Rb pathways, coupled by the accumulation of CDK inhibitors as p21 (Roninson, 2002; Holst et al., 2003). In this study, we have evaluated KLF6 involvement in the senescence process triggered by two different stimuli: *i-* an oncogenic stress attained by the expression of a constitutively active Ras form (H-Ras^G12V^) under the control of a tetracycline responsive promoter (0.1–1.0 μg/ml for 6 days) and *ii-* oxidative treatment of cells with H_2_O_2_, as described previously (Volonte et al., 2002). H-Ras expression was confirmed by immunoblotting (Figure 1A). By SA-β-Gal activity determination, a significant increase in the index of cellular senescence was detected in murine fibroblasts NIH3T3 after 6 days either in response to H-Ras^G12V^ expression (46 ± 6 and 40 ± 6%, dose, respectively, *p* < 0.05, Figure 1B) or H_2_O_2_ treatment (66 ± 7%, *p* < 0.05, Figure 2A). Tetracycline treatment, *per se*, did not lead to significant changes in the cellular senescence index nor increments in KLF6 protein expression (Supplementary Figures 1A,B). Both senescence phenotypes were accompanied by increments in p21 expression levels and hallmarks of DNA damage response activation, as highlighted by immunostaining of p21, γ-H2AX, pATM, and p53 protein levels (Figures 1C, 2C). In addition, each stimulus also increased the apoptosis rate, as assessed by Annexin V/propidium iodide staining (*p* < 0.05, Supplementary Figures 2B,D). The splice variants were not analyzed due to KLF6 splicing has not been described in mouse. Moreover, oxidative-induced senescence correlated with a slower proliferation rate (*p* < 0.05, Supplementary Figure 2A), while oncogenic H-Ras^G12V^ expression shows an increase in the relative cell number (*p* < 0.05, Supplementary Figure 2C), as it has been previously reported (Trucco et al., 2014). Notably, both oncogene and oxidative-induced cellular senescence processes were accompanied by increased KLF6 protein expression (Figures 1A, 2B, respectively), showing different timepoints profile (Supplementary Figures 1E–G), thus supporting a potential association of KLF6 with cellular senescence modulation in response to different triggers. Moreover, the H-Ras^G12V^ oncogene stimulus showed an increase in KLF6 mRNA levels, as previously reported (Trucco et al., 2014), although this effect could not be detected for H_2_O_2_ treatment (Supplementary Figures 1H–J).

### KLF6 Silencing Impairs Oxidative and Oncogene-Induced Cellular Senescence

**FIGURE 3:**
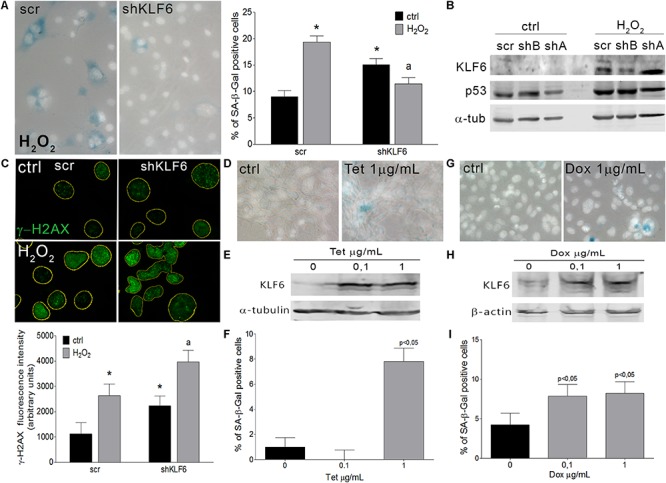
Krüppel-like factor 6 silencing interferes with oxidative-induced cellular senescence. **(A)** Left: Representative micrograph of murine NIH3T3 fibroblasts stably transduced to express either scramble (scr) or KLF6-specific shRNA (shKLF6) were treated with H_2_O_2_ (150 μM for 2 h) and processed after 6 days to detect SA-β-Gal staining (cytoplasmic blue stain). Nuclear fluorescent dye Hoechst was applied to denote cell nuclei (gray stain). Images were captured at X400 magnification and are representative of three independent experiments. Right: Cellular senescence index expressed as the percentage of SA-β-Gal positive cells. **(B)** KLF6 immunoblotting from murine NIH3T3 fibroblasts expressing scramble or KLF6-specific shRNA after 4 h of H_2_O_2_ treatment. Anti-α-tubulin was used as loading control. Images are representative of three independent experiments. **(C)** Upper: Representative micrograph of DNA damage biomarker γ-H2AX in murine fibroblast by fluorescence immunodetection. Nuclei area is represented by yellow contours extracted from Hoechst fluorescence staining. Images are representative of three independent experiments. Lower: Fluorescence intensity of γ-H2AX antibody fluorochrome emission was evaluated in five random chosen fields and normalized by total DNA content area. Two-way ANOVA was used to examine the effect of shKLF6 and H_2_O_2_ in three independent samples. **(D)** Representative micrograph of murine NIH3T3 fibroblasts stably transduced to express KLF6 under the control of a tetracycline-inducible promoter were treated with tetracycline (0.1 and 1.0 μg/mL Sigma-Aldrich) during 6 days and processed to detect SA-β-Gal staining. Nuclear fluorescent dye Hoechst was applied to denote cell nuclei (gray stain), X400 magnification. **(E)** KLF6 immunoblotting from murine NIH3T3 fibroblasts expressing KLF6 after tetracycline treatment. Anti-α-tubulin was used as loading control. Images are representative of three independent experiments. **(F)** Cellular senescence index expressed as the percentage of SA-β-Gal positive cells in NIH3T3 fibroblasts expressing KLF6. **(G)** Representative micrograph of human cervix cancer HeLa cells stably modify to express KLF6 under the control of a doxycycline-inducible promoter were treated with 0.1 and 1.0 μg/mL of doxycycline (Sigma-Aldrich) during 6 days and processed to detect SA-β-Gal staining. Nuclear fluorescent dye Hoechst was applied to denote cell nuclei (gray stain), X400 magnification. **(H)** KLF6 immunoblotting from HeLa cells expressing KLF6 after doxycycline treatment. Mouse anti-β-actin (Sigma-Aldrich) was used as loading control. Images are representative of three independent experiments. **(I)** Cellular senescence index expressed as the percentage of SA-β-Gal positive cells in HeLa cells expressing KLF6 under doxycycline control. Data are presented as media ± SEM. Fisher Test was applied to detect significant difference, *p* < 0.05. Asterisk (^∗^), “a” indicate means significantly different.

**FIGURE 4:**
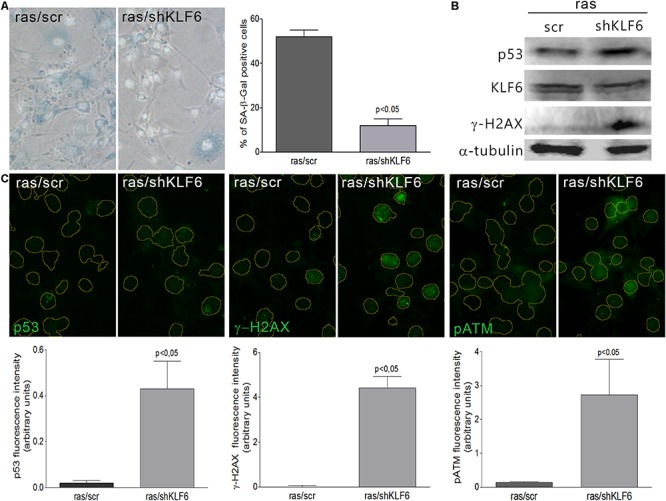
Krüppel-like factor 6 silencing interferes with oncogene-induced cellular senescence. **(A)** Left: Representative micrograph of murine NIH3T3 fibroblasts stably transduced to express a constitutively active form of H-Ras (H-Ras^G12V^) and either scramble (ras/scr) or KLF6-specific shRNA (ras/shKLF6), cultured during 6 days and processed to detect SA-β-Gal staining (cytoplasmic blue stain). Nuclear fluorescent dye Hoechst was applied to denote cell nuclei (gray stain). Images were captured X400 magnification. Right: Cellular senescence index expressed as the percentage of SA-β-Gal positive cells. **(B)** Immunoblotting for KLF6, p53 and γ-H2AX from murine NIH3T3 fibroblasts expressing H-Ras^G12V^ and scramble or shKLF6. Anti-α-tubulin was used as loading control. Images are representative of three independent experiments. **(C)** Upper: Representative micrograph of DNA damage response biomarkers: p53, γ-H2AX and phospho ATM by fluorescence immunodetection on murine NIH3T3 fibroblasts expressing H-Ras^G12V^ and scramble or shKLF6. Lower: Fluorescence intensity of each antibody fluorochrome emission was evaluated in five random chosen fields and normalized by total DNA content area. Data are presented as media ± SEM. Fisher Test was applied to detect significant difference, *p* < 0.05.

To verify whether KLF6 is involved in cellular senescence induction, we down-regulated endogenous KLF6 levels by stable expression of a KLF6 shRNA in NIH3T3 fibroblasts (shKLF6). Cells were then treated with H_2_O_2_ as described above, or left untreated in fibroblasts that constitutively express the active form of the H-Ras^G12V^ oncogene (ras/shKLF6). Two different shKLF6 sequences were assayed and KLF6 down-regulation was corroborated by immunoblotting (Figure 3B). The shRNA sequence that led to more effective KLF6 silencing was chosen for further experiments (Figures 3B, 4B). KLF6 down-regulation resulted in the attenuation of both oxidative and oncogene stimuli ability to induce cellular senescence, as was determined by a significant decrease in the rate of SA-β-Gal positive cells (*p* < 0.05, Figures 3A, 4A). These findings suggest that cellular senescence might be in part dependent on KLF6 function.

Krüppel-like factor 6 silencing in both senescent contexts also induced DNA damage markers, evaluated by immunodetection of γ-H2AX, pATM, and p53 proteins (*p* < 0.05, Figures 3B,C, 4B,C), showed an increased number of apoptotic cells accordingly to cell cytometer analysis (*p* < 0.05, Supplementary Figures 2F,H) without modifications in the total cell number (Supplementary Figures 2E,G). Moreover, it was observed that decreased KLF6 expression, by itself, raised the basal cellular senescence index (*p* < 0.05, Figure 3A), while increasing cell proliferation rate (*p* < 0.05, Supplementary Figure 2E) and γ-H2AX and p53 levels (*p* < 0.05, Figures 3B,C). Interestingly, we also detected an elevated number of micronuclei, a hallmark of genomic instability in KLF6-silenced fibroblasts (*p* < 0.05, Supplementary Figures 2I,J). Taken together, these findings suggest that KLF6 may contribute to DNA stability maintenance as well as the modulation of apoptosis in the presence of oxidants stressors and oncogenic stimuli.

### Ectopic Expression of KLF6 Induces Cellular Senescence in Normal and Tumoral Contexts

To further explore KLF6 biological activity during the induction of cellular senescence, we generated an NIH3T3 stable cell line in which KLF6 expression is regulated by a tetracycline responsive promoter (0.1–1.0 μg/ml for 6 days, Figure 3E). The quantification of SA-β-Gal positive cells showed that the ectopic expression of KLF6 was able to increase the cellular senescence index (7.80 ± 1.08% *p* < 0.05, Figures 3D,F). These data suggest that in normal contexts, without any specific damage or stressful stimuli, KLF6 expression can trigger cellular senescence. Also, to evaluate whether such KLF6 role can be extended to tumoral contexts, we determined the rate of cellular senescence in the cervical carcinoma HeLa cell line, which was reported to express low levels of endogenous KLF6 protein (Gehrau et al., 2011). To this end we controlled KLF6 expression with doxycycline responsive promoter (0.1–1.0 μg/ml for 6 days, Figure 3H). Doxycycline treatment, *per se*, did not lead to significant changes in the HeLa cellular senescence index nor increments in KLF6 protein expression (Supplementary Figures 1C,D). Strikingly, increased expression of KLF6 in this cell line was able to induce a significant increase in the proportion of SA-β-Gal cells positive (7.86 ± 1.47 and 8.20 ± 1.47% doses, respectively, *p* < 0.05, Figures 3G,I), suggesting that KLF6 tumor suppressor role could be related to the induction of the cellular senescence.

## Discussion

An intricate interaction between diverse and divergent signaling pathways governs the tumor cells ability to proliferate, survive, and invade. KLFs family members have been associated to carcinogenesis, either counteracting or cooperating with tumor development (Tetreault et al., 2013). KLF6 function in the regulation of tumor growth has been validated through several approaches. KLF6 expression attenuates the tumorigenicity of glioblastoma multiforme cells both *in vitro* and *in vivo* and is able to inhibit cellular transformation induced by a variety of oncogenes (Kimmelman et al., 2004). Also, the upregulation of KLF6 significantly reduces cell proliferation in prostate cancer (Narla et al., 2001; Narla et al., 2005; Narla et al., 2007) and in non-small cell lung cancer in a similar way as p53 does (Ito et al., 2004). Furthermore, previous results from our laboratory showed that KLF6 down-regulation predisposes to spontaneous cellular transformation and increases the tumorigenic potential of NIH3T3 cells, associated with a reduced expression of p21^Waf/Cip1^ (Trucco et al., 2014). Additionally, KLF6 interferes with the oncogenic signaling and transformation triggered by activated H-Ras (Trucco et al., 2014).

The precise underlying mechanism by which KLF6 exerts its tumor suppressor role involves a variety of complex pathways. It have been described the transactivation of p21^Waf/Cip1^ in a p53-independent manner, reduction of cyclin D1/cdk4 complexes through interaction with cyclin D1, inhibition of c-Jun proto-oncoprotein activities, decreased VEGF expression and apoptosis induction (Benzeno et al., 2004; Slavin et al., 2004; Racca et al., 2016). However, the potential association of KLF6 with cellular senescence has not been thoroughly defined so far. To date, some reports have described that KLF6 overexpression results in the upregulation of differentiation markers associated to a cell proliferation inhibition and cellular senescence in liposarcoma (Keung et al., 2015) and glioblastoma (Masilamani et al., 2017).

The induction of senescence involves multiple signals and genes depending on the cell context, indicating the intricate nature of this phenotype (Campisi and d’Adda di Fagagna, 2007; Courtois-Cox et al., 2008; d’Adda di Fagagna, 2008). Frequently, cells with high levels of DNA damage can trigger cellular senescence and steadily lose their ability to undergo cell proliferation (Campisi, 2013). Beyond the typical DNA damage, oncogene activation or DNA replication stress, like the one elicited by oncogenic mutated Ras (Serrano et al., 1997), is likely to result in reactive oxygen species or in DNA hyper-replication, leading to an activation of the DNA damage response, p53 activation and consequently, converging in senescence induction (Di Micco et al., 2006; Halazonetis et al., 2008; Pospelova et al., 2009). This response might prevent genomic instability accumulation, thus suggesting that senescence acts as a barrier to cellular transformation. Elucidating the molecular mechanisms which overrule the cellular responses elicited by genotoxic stimuli or aberrant proliferation is still a challenge in the tumor growth field. Herein, we expose the association of KLF6 up-regulation in two different cellular senescence scenarios. We also observed that KLF6 silencing bypassed either oxidative or oncogene-induced senescence. Moreover, KLF6 expression *per se* was capable to trigger cellular senescence in both normal and tumoral contexts. Together, these results provide insights into the mechanism by which KLF6 plays a suppressing role of uncontrolled or damaged cell proliferation.

Since KLF6 signaling pathways are assumed to be very complex and diverse, the exact mechanism by which KLF6 participate in cellular senescence will require further investigation. Nonetheless, we position KLF6 as novel factor capable to induce cellular senescence and a necessary player for successful senescence establishment (Figures 3A, 4A). This new role could be related to its known ability to block cell cycle progression through the regulation of its specific target genes (e.g., p21^Waf/Cip1^, cyclin D1, c-Jun, c-Myc, and p53) (Andreoli et al., 2010). Although other mechanisms cannot be excluded, such as the transforming growth factor β (TGFβ) pathway (Kojima et al., 2000), which is a well-established KLF6 target gene that was already associated to cellular senescence induction (Frippiat et al., 2001; Senturk et al., 2010), making plausible for both transcription factors to cooperate in the senescence process (Kojima et al., 2000; Botella et al., 2009; Dionyssiou et al., 2013). Also, a biological KLF6 cross-talk with p53 could be considered since several independent reports proposed that a significant portion of the biological actions of KLF6 may result from protein-protein interactions and the extent on the p53 cellular status (Rubinstein et al., 2004; Andreoli et al., 2010; Gehrau et al., 2011), pointing KLF6 as a potential p53 partner that contribute to determine cell fate decisions.

At the end of our study, based on the results in the NIH3T3 model, we overexpressed wild type KLF6 in HeLa cell line derived from human cervical carcinoma, but we did not overexpress any of the splice forms since they are not subject of study in the present brief report. Furthermore, previous works suggested that the SV1 variant may not have a leading role in the induction of cellular senescence in other human tumor models. In this sense, it has been shown that prolonged expression of KLF6-wt, but not KLF6-sv1, induced a senescent-like phenotype in two cell lines derived from glioblastoma: LN229 (glioblastoma cell line) and BTSC23 (Masilamani et al., 2017). Moreover, it was reported that KLF6 acts as a tumor suppressor in liposarcoma inhibiting cellular proliferation and invasion, and drive senescence and differentiation through the regulation of master regulators of adipogenesis (Keung et al., 2015).

Krüppel-like factor 6 is also considered a stress response factor as its expression is exhaustively regulated by several cell-damaging stimuli (Botella et al., 2002; Warke et al., 2003; Kiang et al., 2004; Slavin et al., 2004; Tahara et al., 2009; Gehrau et al., 2011). In the present study, both oncogenic and oxidative stress stimuli were accompanied by the upregulation of KLF6, similarly to prior reports (Gehrau et al., 2011; Urtasun et al., 2012; Trucco et al., 2014). Surprisingly, KLF6 silencing *per se* showed DNA damage accumulation, increased p53 expression and also displayed signs of genomic instability (Figures 3B,C and Supplementary Figures 2I,J), which could contribute to explain the bias for spontaneous cellular transformation previously described for this phenotype (Trucco et al., 2014). KLF6 down-regulation also led to a rise in the basal index of cellular senescence and, despite harboring a relatively high level of p53 that could operate to avoid DNA damage, KLF6-silenced cells still exhibit genetic instability features (Supplementary Figures 2I,J). This indicates that KLF6 activity could safeguard DNA stability, as reported for KLF4 (Hagos et al., 2009). Thus, these findings put forward a potential role for KLF6 in cellular housekeeping programs that pursue the control of normal proliferation and genome maintenance.

Cellular stress and damaging stimuli can lead to senescence or apoptosis and even both phenotypes simultaneously (Chen et al., 2000). Recently, relevant molecular mechanistic insights have appeared with the aim of understanding the relationship between both cell fates (Childs et al., 2014). In this study, we observed that senescence outcome yielded priority in both oxidative and oncogene stress stimuli models. However, KLF6 silencing enhanced the DNA damage and genomic instability signals gathered after stress exposure, which in turns was coupled by an increased apoptotic rate whereas cells failed to achieve senescence status. Seemingly, KLF6 down-regulation could favor DNA damage accumulation and predispose to cell death.

Several mechanisms could converge to determine cell fate between senescence and apoptosis after genotoxic stress, as the extent and duration of DNA damage (Stein et al., 1999; Rodriguez and Meuth, 2006). On its regard, KLF6 expression seems to be regulated by the amount of DNA damage since previous reports denoted that low doses of DNA-damaging agent did not affect KLF6 expression while high doses compatible with apoptosis induction, ended in a down-regulation of its levels (Banck et al., 2006; D’Astolfo et al., 2008). In fact, in line with the results presented here, KLF6 knockdown in hepatocarcinoma cells render cells more susceptible to DNA-damage induced apoptosis, indicating that endogenous KLF6 may act at some level blocking apoptosis entry (Sirach et al., 2007; D’Astolfo et al., 2008). Moreover, KLF6 expression in Ras-transformed and tumoral HeLa cells shows to confer a cell death resistance upon treatment with DNA-damaging agents (D’Astolfo et al., 2008; Trucco et al., 2014). Thus, in a context of genotoxic stress, KLF6 may favor apoptosis blockage and, direct or indirectly, participate in senescence induction. This observation raises the possibility that KLF6 could engage senescence and apoptosis pathways in certain processes or stress responses.

In conclusion, based on the results presented herein, we suggest that KLF6 may be in part necessary in the induction of the cellular senescence program in response to cell damage stimuli such as oncogenic and oxidative stress. Our data provide insights into the potential mechanism by which KLF6 may play a role in suppressing DNA damage accumulation as well as in oxidative stress responses, and implicate KLF6 in genome maintenance and protection against aberrant cell proliferation. These findings helps to strengthen the understanding of KLF6 function in cellular growth contexts rendering possible for its tumor suppressor activity to be mediated by cellular senescence, representing an alarm sign in response to certain stimuli that lead to an exacerbated proliferation or cell transformation.

## Data Availability Statement

The raw data supporting the conclusions of this manuscript will be made available by the authors, without undue reservation, to any qualified researcher.

## Author Contributions

MS, AC, AR, SC, MP, and GS made substantial contributions to the conception and design, and/or acquisition of the data, and/or analysis and interpretation of the data. MS, GS, and JB participated in drafting the manuscript or revising it critically for important intellectual content. All authors gave the final approval of the version to be submitted and any revised version.

## Conflict of Interest

The authors declare that the research was conducted in the absence of any commercial or financial relationships that could be construed as a potential conflict of interest.
