# Supplementary figures and images for: Krüppel-Like Factor 6 Is Required for Oxidative and Oncogene-Induced Cellular Senescence
Source: Front Cell Dev Biol. 2019 Nov 22;7:297. doi: 10.3389/fcell.2019.00297 (PMC6882731; doi:10.3389/fcell.2019.00297)

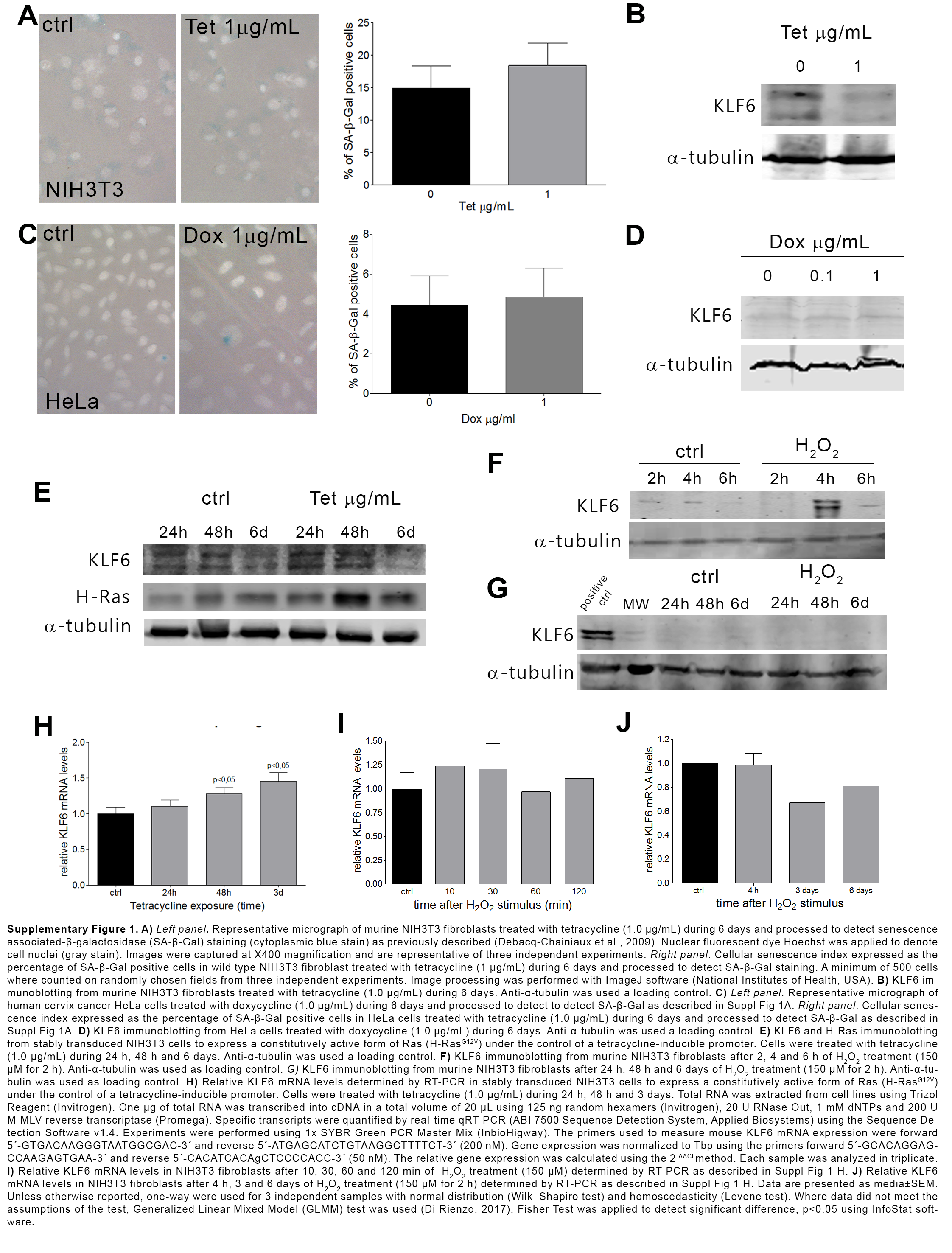

Supplement: Supplementary file 1 [file Image_1.TIF]

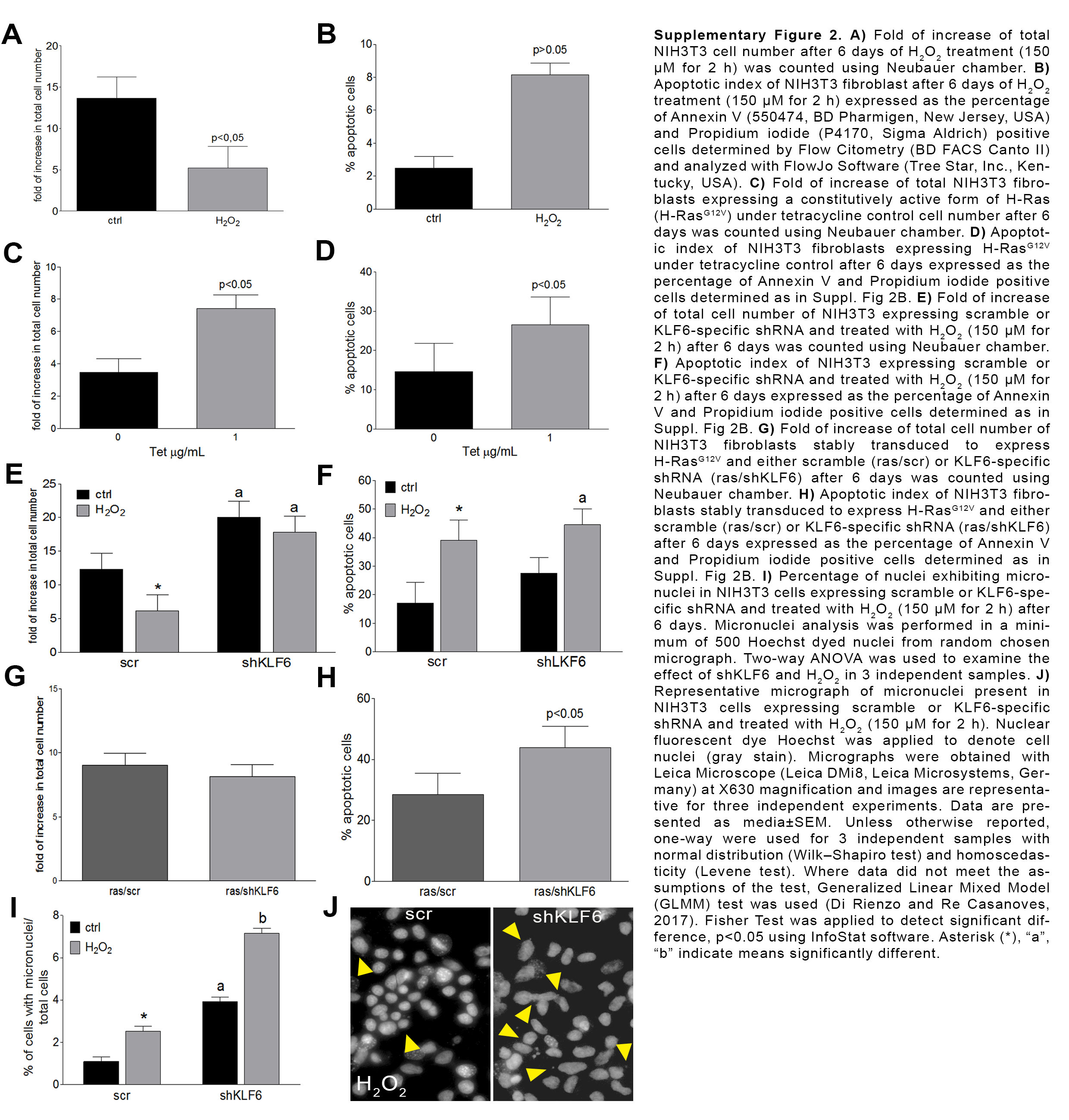

Supplement: Supplementary file 2 [file Image_2.tif]
